# Frog Skin Antimicrobial Peptide 3-13 and Its Analogs Alleviate Atherosclerosis Cholesterol Accumulation in Foam Cells via PPARγ Signaling Pathway

**DOI:** 10.3390/cells14181470

**Published:** 2025-09-19

**Authors:** Xue-Feng Yang, Zi-Meng Hao, Xin-Yu Cui, Wan-Qi Liu, Meng-Miao Li, De-Jing Shang

**Affiliations:** 1 School of Life Science, Liaoning Provincial Key Laboratory of Biotechnology and Drug Discovery, Liaoning Normal University, Dalian 116029, China; yangxuefeng@jzmu.edu.cn (X.-F.Y.); haozimeng_lnnu@hotmail.com (Z.-M.H.); cuixinyu_lnnu@hotmail.com (X.-Y.C.); liuwanqi_lnnu@hotmail.com (W.-Q.L.); 2 School of Basic Medical Sciences, Department of Physiology, Jinzhou Medical University, Jinzhou 121000, China

**Keywords:** frog skin antimicrobial peptide, atherosclerosis, lipid metabolism, PPARγ

## Abstract

Atherosclerosis (AS), a major contributor to cardiovascular disease, hypertension, and stroke, is associated with significant morbidity and mortality. Antimicrobial peptides (AMPs) 3-13, W3R6, and chensinin-1b were engineered based on the sequence of chensinin-1, originally isolated from the skin secretion of *Rana chensinensis*. This study investigated their therapeutic potential in *ApoE^-/-^* AS mice and THP-1-derived foam cells, focusing on the regulation of cholesterol metabolism. AMP 3-13 markedly reduced body weight gain, aortic root plaque formation, and plasma cholesterol levels in *ApoE^-/-^* mice. Transcriptomic analysis revealed that AMP 3-13 significantly altered gene expression related to cholesterol metabolism and the PPAR signaling pathway. Specifically, AMP 3-13 upregulated PPARγ, ABCA1, and ABCG1, while downregulating CD36 in aortic root plaques. In THP-1-derived foam cells, AMP 3-13 and its analogs activated the PPARγ–ABCA1/ABCG1 axis, enhancing cholesterol efflux. Concurrently, they inhibited CD36 expression by competing with PPARγ for promoter binding, thereby reducing ox-LDL uptake. These findings suggested that AMP 3-13 and its analogs represented promising therapeutic agents for AS through their ability to reduce cholesterol accumulation in foam cell.

## 1. Introduction

Atherosclerosis (AS) is a chronic disease characterized by plaque accumulation within the arterial walls, resulting in severe cardiovascular events such as heart attack and stroke [1]. Foam cell formation is a distinguished feature of AS and contributes to the formation of fatty streaks, which are the initial visible indication of AS [2,3,4]. The process begins with abnormal lipid metabolism and the accumulation of low-density lipoprotein (LDL) in the arterial walls [5]. Next, LDL interacts with oxidizing agents such as free radicals or reactive oxygen species, which initiate oxidation reactions and lead to the formation of oxidized low-density lipoprotein (ox-LDL) [6]. The deposition of ox-LDL triggers the release of proinflammatory factors and promotes monocyte differentiation into macrophages, which further develop into foam cells through the regulation of ox-LDL uptake and cholesterol efflux [7,8]. Peroxisome proliferator-activated receptor γ (PPARγ) is a critical transcription factor to regulate ox-LDL uptake and cholesterol efflux in foam cells [9]. The activation of PPARγ upregulates ATP binding box transporter A1/G1 (ABCA1/ABCG1) expression, thereby promoting cellular cholesterol efflux [10]. In addition, PPARγ binds with RXRα to form heterodimers, which interact with the peroxisome proliferator-responsive element (PPRE) motif in the cluster of differentiation 36 (CD36) promoter, leading to ox-LDL uptake [11,12]. Targeting PPARγ to regulate cholesterol uptake and efflux is an important strategy for treating AS [13].

Statins are typically the first choice for the treatment of AS due to their ability to lower low-density lipoprotein cholesterol (LDL-C) levels, reduce inflammatory responses, and stabilize atherosclerotic plaques [14,15]. Although statins display considerable efficacy and wide applicability, their side effects, such as muscle soreness and hepatotoxicity, cause numerous patients to cease therapy even if their lipid levels fail to meet the recommended guidelines for ceasing treatment [16]. Rosiglitazone (RSG), a type of thiazolidinedione (TZD) that activates PPARγ and upregulates ABCA1 and ABCG1 expression, thereby increasing cholesterol efflux from macrophages [17,18]. However, it should be used cautiously because of the risk of mild anemia and the potential for increased heart failure [19]. In recent years, several novel drugs that target lipid metabolism have been approved for the treatment of AS. For example, alirocumab (AOC) and evolocumab (EVO), two monoclonal antibodies that inhibit PCSK9, increase the expression of LDL receptors on the surface of hepatocytes, thereby reducing LDL-C levels [20,21]. Although monoclonal antibody drugs are highly effective and precise, they are expensive and may cause immune reactions [22]. Therefore, developing safer, more effective and cost-efficient alternative therapies is crucial.

Antimicrobial peptides (AMPs), a subclass of bioactive peptides, are evolutionarily conserved defense molecules produced by all living organisms, typically comprising 12–50 amino acids [23]. AMPs can be classified based on their biological sources, structural features, and target microorganisms [23]. Based on biological sources, they are categorized as animal-derived, plant-derived, microbial-derived, or synthetic/engineered variants. Structurally, these peptides adopt distinct conformations including α-helical, β-sheet, extended/linear, and cyclic architectures. Functionally, AMPs are differentiated into antibacterial, antifungal, antiviral, and broad-spectrum subgroups. Unlike other bioactive peptides, AMPs are traditionally recognized for their direct antibacterial activities via membrane disruption [24].However, recent research has uncovered unconventional roles of AMPs in: modulating immune responses (e.g., human cathelicidin LL-37) [25,26], promoting wound repair (e.g., β-defensin) [27], and regulating metabolic pathways (e.g., adipocetin) [28].

Recent studies have explored the potential of AMPs in combating AS because of their ability to modulate lipid metabolism and reduce inflammation [29]. LL-37 is a cathelicidin-like AMP found in humans that is expressed predominantly in macrophages, and the levels of LL-37 are higher in atherosclerotic lesions than in normal blood vessels [30]. A recent study reported that LL-37 inhibits cholesterol accumulation by decreasing the expression of CD36 through the ERK signaling pathway [31]. Additionally, PR-39 is a natural AMP that has cardioprotective effects by inhibiting inflammation through inactivating the proteasome-mediated degradation of IκBα [31].

Our previous research revealed that the frog skin AMP temporin-1CEa and its analogs exhibited anti-atherosclerotic effects by reducing the formation of macrophage-derived foam cells [32]. The frog skin AMP chensinin-1b was designed in our lab by replacing the hydrophobic and hydrophilic residues on the opposite side of the parent peptide chensinin-1 [33]. Moreover, AMP 3-13 was synthesized by truncating positions 3 to 13 of chensinin-1b, and AMP W3R6 was obtained by replacing the His residues at positions 4 and 10 with Arg residues of AMP 3-13 [34,35]. Our previous study has revealed the antibacterial activity and the structural characteristics of the AMPs [34]. All of the AMPs exhibited a relatively high positive charge density. Notably, the NMR structure of chensinin-1b revealed a partially α-helical region (residues 8–14) in a membrane-mimetic environment. In comparison, AMP 3-13 demonstrated a higher α-helical content than chensinin-1b [34]. In this study, *ApoE^-/-^* mice were fed a high-fat diet (HFD) to establish an AS mouse model, and THP-1 cells were induced with ox-LDL to establish foam cell model. The effects of AMP 3-13 and its analogs, particularly AMP 3-13, on *ApoE^-/-^* AS mice and foam cell formation were investigated. In addition, the underlying mechanism of how ox-LDL uptake and cholesterol efflux are regulated in foam cells was elucidated. This study provides novel insights into the potential of 3-13 and its analogs as therapeutic agents for the prevention and treatment of AS.

## 2. Materials and Methods

### 2.1. Reagents

ox-LDL was purchased from Yiyuan Biotechnology Co., Ltd. (Guangzhou, China). The frog skin peptides chensinin-1b, W3R6 and 3-13 were synthesized by GL Biochemistry, Inc. (Shanghai, China), and the purity of the peptides was greater than 95%. The amino acid sequences and physicochemical information of chensinin-1b, W3R6 and 3-13 are summarized in Table S1. RSG and T0070907 was purchased from MedChemExpress Co., Ltd. (Shanghai, China), and dissolved in DMSO (solubilities 175 and 62.5 mg/mL).

### 2.2. ApoE^-/-^ Mouse Model of AS

All experimental protocols were approved by the Medical Ethics Committee of Liaoning Normal University. *ApoE^-/-^* mice were purchased from Vital River Laboratory Animal Technology (Beijing, China). *ApoE^-/-^* mice were 6 weeks old and maintained on a SPF C57BL/6 background. *ApoE^-/-^* mice were identified at 4 weeks after birth. The mouse tail genotype was identified by using a mouse genotyping kit, and *ApoE^-/-^* homozygous mice were used in subsequent experiments. *ApoE^-/-^* female mice aged 6 weeks were fed a HFD to establish an AS mouse model. After the HFD was continued for 6 weeks, the mice were randomly divided into the ctrl, HFD and HFD+AMP 3-13 groups, with 10 mice in each group. The AMP 3-13 were dissolved in 0.9% saline and administered intraperitoneally daily for 14 days. The body weight, activity and mental status of the mice in each group were observed and recorded every day.

### 2.3. Aorta Oil Red O (ORO) Staining

After anesthesia, plasma was collected from the mice in each group. The abdominal cavity and thoracic cavity of each mouse were cut open, the excess tissue around the aortic vessels was removed to expose the intact aortic vessels, and the perivascular adipose tissue was carefully removed. The whole aortic vessel was isolated by clipping from the proximal end, along the spine of the mouse to the iliac artery, with small spring-loaded scissors. The scissors were inserted into the proximal end of the aorta, and the aortic arch, non-aorta, left common carotid artery, the left subclavian artery, the thoracic aorta, the abdominal aorta and the left and right iliac arteries were harvested along the inner curvature of the aortic arch. Finally, the whole blood vessels were isolated. Staining was performed with an ORO staining kit (G1260, Solarbio, Shanghai, China).

### 2.4. Histological Detection

The aortic vessel was fixed in 4% paraformaldehyde in phosphate-buffered saline and embedded in paraffin. The paraffin-embedded tissue was cut into 4 μm-thick sections. The sections were stained with hematoxylin and eosin (HE, Sigma-Aldrich, St. Louis, MO, USA).

### 2.5. Plasma Lipid Factor Assay

After 14 days, the mice in each group were anesthetized, and the eyeball blood was collected in an anticoagulant tube containing EDTA. The whole blood was mixed with the anticoagulant thoroughly and centrifuged at 3000 rpm for 10 min, and the resulting mixture was stored at −80 °C. Total cholesterol (T-CHO), LDL-C, triglyceride (TG) and high-density lipoprotein cholesterol (HDL-C) kits from Nanjing Jian Cheng Bioengineering Institute Co., Ltd. (Item No.: A111-1-1, A113-1-1, A110-1-1 and A112-1-1) were used to detect blood lipid parameters. The detection ranges of the kits are 0–19.39, 0–10.40, 0.30–11.40 and 0–5.16 mmol/L, respectively. The sensitivities are 2.00 mmol/L (absorbance 0.10–0.40), 2.60 mmol/L (absorbance 0.18–0.28), 2.70 mmol/L (absorbance 0.20–0.40) and 1.00 mmol/L (absorbance > 0.04), respectively. The results were obtained using Multiskan FC microplate reader (Thermo Fisher Scientific, Waltham, MA, USA).

### 2.6. RNA-Seq Assay

HFD-induced *ApoE^-/-^* AS mice were treated with AMP 3-13 as previously described. The mice were randomly divided into the following groups: the ctrl, HFD and HFD+3-13 groups with three replicates in each group. The mice in each group were anesthetized with sodium pentobarbital (50 mg/kg I.P), the abdominal cavity was washed with about 1 to 2 mL cold PBS containing 10 mM EDTA, the peritoneal exudate was collected and centrifuged at 400 g for 10 min. The cell pellets were washed twice with PBS and then resuspended in 0.2 mL DMEM to count the cells. The isolated macrophages were plated separately in 6-well tissue culture plates and cultured with RPMI/10% FBS overnight. The next morning, nonadherent cells were removed by aspiration, adherent cells (macrophages) were washed with PBS three times and harvested for the experiments. 600 μL Trizol lysate was added to collect the cells, and then the cells were frozen in liquid nitrogen for 20 min and transferred to the −80 °C refrigerator for storage. Samples were sent to the company for RNA-seq analysis.

Total RNA purity and concentration were assessed using a NanoDrop 2000 spectrophotometer (Thermo Fisher Scientific, Waltham, MA, USA). RNA integrity was verified with an Agilent 2100 Bioanalyzer (Agilent Technologies, Santa Clara, CA, USA), where all samples met the following quality thresholds: RNA Integrity Number (RIN) ≥ 8.0, OD_260/280_ > 1.9 and Total RNA > 500 ng. Sequencing libraries were constructed from 1 μg qualified RNA using the VAHTS Universal V6 RNA-seq Library Prep Kit (Vazyme Biotech, Nanjing, China) according to manufacturer protocols. The transcriptome sequencing and analysis were conducted by OE Biotech Co., Ltd. (Shanghai, China). Library sequencing was performed on an Illumina NovaSeq 6000 platform (Illumina Inc., San Diego, CA, USA) to generate 150 bp paired-end reads. Adaptor trimming and low-quality read removal using fastp (v0.23.2) [36]. Clean reads were mapped to the *Mus musculus* reference genome (Genome assembly GRCm39) using HISAT2 (v2.2.1) [37] with default parameters. Gene-level counts were generated by HTSeq-count (v0.13.5) [38]. Differentially expressed genes (DEGs) were identified using DESeq2 (v1.30.1) [39] with the following criteria: |log_2_ (fold change)| > 1 and Benjamini–Hochberg adjusted *p*-value < 0.05. Hierarchical clustering of DEGs was performed in R (v4.2.0) using Euclidean distance with complete linkage. Volcano plot and heatmaps generated with bioinformatics platform, and WikiPathways enrichment bubble plots created using ggplot2 (v3.3.5). The raw RNA-seq data has been submitted to the NCBI SRA datasets with accession number PRJNA1290911.

### 2.7. Cell Culture

The human leukemia monocytic cell line THP-1 was purchased from Jiangsu Kaiji Biology Co., Ltd. (Nanjing, China). The cells were cultured in RPMI 1640 medium supplemented with 10% FBS and 1% penicillin and streptomycin. Next, THP-1 cells were induced to differentiate into macrophages by incubation with 100 ng/mL phorbol 12-myristate 13-acetate (PMA, MedChemExpress Co., Ltd., Shanghai, China) for 48 h, during which the cells adhered to the culture surface. Finally, to synchronize the macrophages, the medium was replaced with serum-free RPMI 1640 medium and the cells were cultured for an additional 24 h. The in vitro assays were divided into the control group (ctrl, THP-1 cells), ox-LDL group (ox-LDL, 100 μg/mL), PPARγ agonist group (ox-LDL+RSG, 20 μM), PPARγ inhibitor group (ox-LDL+T0070907, 10 μM) and AMPs groups (ox-LDL+3-13 or its analogs, 6.25, 12.5 and 25 μM).

### 2.8. Quantitative Real-Time PCR

The samples of aortic arch tissue in each group were the same as before. Macrophages were seeded in 6-well plates and then treated with ox-LDL, 3-13 and its analogs for an additional 24 h. RSG (20 μM) was added to the PPARγ agonist group. The total RNA of aortic arch tissue and macrophages were extracted with TRIzol reagent (15596018CN; Invitrogen, Shanghai, China). Reverse transcription was performed via a Super Script™ III kit (18080400, Invitrogen, Shanghai, China). cDNA amplification was performed in an ABI Prism 7500 Fast sequence detection system (Applied Biosystems, San Francisco, CA, USA) using a TB Green Premix Ex Taq II kit (RR820Q, TaKaRa, Dalian, China). The primer sequences are summarized in Table S2. The relative mRNA expression levels were normalized to that of GAPDH (internal control) by using the Ct values calculated according to the manufacturer’s instructions.

### 2.9. Western Blot Analysis

The samples of aortic arch tissue in each group were the same as before. Macrophages were seeded in 6-well plates and then treated with drugs in each group. The aortic arch tissue and macrophages in each group were lysed in RIPA lysis buffer. After protein quantification using a BCA kit, the protein in the cell lysates was separated via 10–12% SDS–PAGE before being electrotransferred to a PVDF membrane via standard procedures. After being blocked with 5% skim milk in TBST for 1 h at room temperature, the membranes were incubated with specific primary antibodies at 4 °C overnight. The primary antibodies used are summarized in Table S3. The relative protein expression levels were normalized to those of histone H3 (nuclear protein) or ATP1A1 (membrane protein). Then, the samples were incubated with secondary antibodies conjugated to HRP. Bands were visualized with an Azure Biosystems c500 instrument using ECL-Plus detection reagents (Santa Cruz, CA, USA). Densitometric quantification of the protein concentration was performed via Image-Pro Plus software (Version 6.0.0.260).

### 2.10. Immunohistochemical

The aortic vessel sections were described as before. After hydration, sodium citrate antigen repair solution was added to the sections in each group, which were then microwaved at 700 W for 5 min and 250 W for 10 min and cooled to room temperature. Endogenous peroxidase was removed by adding 3% H_2_O_2_, blocking with 5% BSA at room temperature for 1 h, adding primary antibodies and incubating overnight in a wet box at 4 °C. The primary antibodies used are summarized in Table S3. HRP-labeled secondary antibodies were added, and the samples were incubated for 1 h at 37 °C. DAB was added for staining in the dark for 3–10 min. Finally, photographs were taken with a microscope.

### 2.11. Cell Proliferation Assay

The growth rate of the THP-1 cells and the concentration of ox-LDL were determined via real-time cellular analysis (RTCA). Fifty microliters of medium were added to the RTCA plate to determine the response baseline, and then 100 μL of cell suspensions with densities of 5 × 10^4^, 1 × 10^5^, 1.5 × 10^5^ and 2 × 10^5^ cells/mL were added. After 48 h, serum-free 1640 medium was added to the well plate, and the plate was treated for 24 h. ox-LDL concentrations of 50, 100, and 200 μg/mL were added, and the proliferation curves of each group at 24 h were generated via RTCA. In accordance with the results of the RTCA and ox-LDL concentration experiments, 1 × 10^5^ cells/mL was selected as the density of THP-1 cells and 100 μg/mL was selected as the concentration of ox-LDL for the subsequent experiments. THP-1 cells were seeded in 96-well plates at a density of 1 × 10^5^ cells/mL. After differentiation and synchronization, 100 μg/mL ox-LDL was added to each group of cells (ox-LDL was not added to the AMP-only treatment groups). The frog skin peptides chensinin-1b, W3R6 and 3-13 were subsequently added at concentrations of 3.125, 6.25, 12.5, 25, 50 and 100 μM, respectively. After 24 h, 10% final concentration of CCK8 was added to each well, and the samples were incubated for 4 h in the dark. The absorbance of each well was measured with a Multiskan FC microplate reader (Thermo Fisher Scientific, Waltham, MA, USA) at 490 nm.

### 2.12. Cholesterol Detection

Macrophages were seeded in 96-well plates, and the cells in each group were treated with drugs. In accordance with the manufacturer’s instructions (BC1985/BC1895, Solarbio, Shanghai, China), total cholesterol (TC) or free cholesterol (FC) working solution was added, and 20 μL of blank control solution, standard solution or sample mixture was added. Each group of samples was incubated for 15 min at 37 °C. The absorbance of each well was measured with a Multiskan FC microplate reader (Thermo Fisher Scientific, Waltham, MA, USA) at 500 nm. Cellular protein was measured with a BCA protein assay kit according to the manufacturer’s instructions (MA0082, Meilunbio, Dalian, China). TC and FC were normalized to the cellular protein levels. Cholesterol ester (CE) levels were calculated via the following formula: CE = TC − FC.

### 2.13. ORO Staining

Macrophages were seeded in 24-well plates and then treated with ox-LDL and/or 3-13 and its analogs for an additional 24 h. Lipid droplets in ox-LDL-stimulated macrophages were observed via ORO staining (G1262, Solarbio, Shanghai, China). In brief, ORO fixative solution was added, and the samples were fixed for 30 min at room temperature. The cells were then incubated in 60% isopropyl alcohol for 5 min. ORO was added to the wells and incubated for 15 min at room temperature. The cells were then counterstained with Mayer’s hematoxylin. Images were acquired via a light microscope (Leica, Wetzlar, Germany).

### 2.14. Nitrobenzoxadiazole (NBD)-Labeled Cholesterol Detection

After drug treatment, NBD-labeled cholesterol (5 μM), 3-13 and its analogs prepared in RPMI 1640 medium without phenol red were added to the THP-1 cells. After 3 h, the cells were washed with PBS, apolipoprotein A-1 (apoA-1) and HDL at concentrations of 10 μg/mL prepared in phenol red-free RPMI 1640 medium were added, and the cells were cultured for another 4 h. The supernatant of each group was collected, which was the cholesterol effluent. After the THP-1 cells were lysed by the addition of 0.1% Triton X-100, the cell lysates from each group were collected. The fluorescence intensity of each sample was measured by a Multiskan FC microplate reader (Thermo Fisher Scientific, Waltham, MA, USA), the excitation wavelength was 469 nm, and the emission wavelength was 537 nm. The cholesterol efflux rates of each group were calculated.

### 2.15. Dil-ox-LDL Uptake Detection

THP-1 cells were seeded in 12-well plates at a density of 1 × 10^5^ cells/mL. After the differentiation and synchronization of the cells in each group, 40 μg/mL (Dil dye-labeled ox-LDL) Dil-ox-LDL prepared in phenol red-free RPMI 1640 medium was added, and AMPs were added at the same time. After 24 h, the cells in each group were washed three times with PBS, and then Hoechst 33342 was added and incubated for 1 h. The slides were removed and sealed by the addition of 5 μL of anti-fluorescence quenching agent. The slides were photographed under a laser confocal microscope at an excitation wavelength of 554 nm and an emission wavelength of 571 nm for Dil-ox-LDL and at an excitation wavelength of 350 nm and an emission wavelength of 461 nm for Hoechst 33342.

### 2.16. Cellular Localization of AMPs

THP-1 cells were seeded in 12-well plates at a density of 1 × 10^5^ cells/mL. After the differentiation and synchronization of the cells in each group, the FITC-labeled antimicrobial peptide 3-13 and its analogs prepared in phenol red-free RPMI 1640 medium were added. After 6 h, the cells in each group were washed three times with PBS, and then Hoechst 33342 was added and incubated for 1 h. The slides were removed and sealed with 5 μL of anti-fluorescence quenching agent. The slides were photographed under a laser confocal microscope at an excitation between of 490–495 nm and emission wavelength between 520 and 530 nm for FITC-AMPs and at an excitation wavelength of 350 nm and an emission wavelength of 461 nm for Hoechst 33342.

### 2.17. Dual-Luciferase Reporter Gene Assay

Total mRNA was extracted from THP-1 cells, and the *PPARγ* gene was amplified via high-fidelity PCR. The target fragment and vector were digested by a double enzyme, and the gel was recovered, ligated, and transformed into *E.coli* DH5α, which was subsequently seeded in plates with lysogeny broth (LB). Positive clones were screened, and gene sequencing was performed to obtain the PPARγ overexpression plasmid. HEK293T cells were used for plasmid transfection, and the cells were divided into a vector group, a ctrl group and an AMP group. First, the PPARγ overexpression plasmid was transfected. After the transfection efficiency was determined, the transfected cells were subsequently transfected with a *CD36* promoter reporter gene plasmid and an internal control reporter gene plasmid. After 6 h, AMP 3-13 were added and detected with a TransDetect^®^ Double-Luciferase Reporter Assay Kit (FR201, Trans, Beijing, China), and after 12 h, the fluorescence intensities of each group were calculated.

### 2.18. ChIP Assay

Studies have shown that PPARγ forms a heterodimer with RXRα, which regulates the expression of target genes by acting on the PPRE sequence of the target gene promoter region. The sequence feature was TGACCT X TGACCT (X is 1 to 2 bases). First, the promoter region of the human *CD36* gene was searched using the UCSC website (https://genome.ucsc.edu/, accessed on 15 August 2023). Then, using the JASPAR website (https://jaspar.elixir.no/analysis, accessed on 15 August 2023), the PPARγ control *CD36* promoter region locus was determined to be TATGACCTAATGAACTAA. The following ChIP primers were designed according to the PPRE sequence of the *CD36* gene promoter region: F: 5′-CAAAAAGGACAGCACGAGCA-3′, R: 5′-ATGCATTCAAACAACCTTAGAAGT-3′. A ChIP kit was used to detect the effects of 3-13 and its analogs on the binding of PPARγ to the *CD36* promoter in THP-1 foam cells. THP-1 cells were seeded in culture flasks at a density of 1 × 10^5^ cells/mL. After the differentiation and synchronization of the THP-1 cells in each group, ox-LDL, 3-13 and its analogs were added. The cells were divided into negative control, positive control, target protein and input groups according to the instructions of the ChIP kit. The binding of PPARγ to the *CD36* promoter in each group was detected by agarose gel electrophoresis.

### 2.19. Electrophoretic Mobility Shift Assay (EMSA)

The 3′ terminal biotin-labeled PPRE sequence probes were purchased from Sangon Biotech (GS009, Shanghai, China), and the EMSA experiment was performed using the Chemiluminescent EMSA Kit (Beyotime Biotechnology, Shanghai, China) according to the manufacturer’s instructions. The AMPs were incubated with PPRE sequence probes at room temperature. Loading buffer was then added to the reaction mixture, and PAGE electrophoresis was performed at 120 V on a 6% TBE gel. Then, the membrane transfer, ultraviolet crosslinking and blocking reaction of the EMSA gel were carried out successively. Streptavidin-HRP conjugate was added, and the mixture was incubated for 15 min. After washing with buffer, the membrane was incubated in substrate balance buffer for 5 min before ECL development. Bands were visualized with an Azure Biosystems c500 instrument using ECL-Plus detection reagents (Santa Cruz, CA, USA). Densitometric quantification of the protein concentration was performed via Image-Pro Plus software (Version 6.0.0.260).

### 2.20. Statistical Analysis

SPSS 18.0 software (SPSS Inc., Chicago, IL, USA) was used to analyze all the experimental data. The experimental results were analyzed by one-way univariate analysis of variance (ANOVA) by Tukey’s test for multiple corrections between groups. sample sizes and statistical method were described in each figure legend. *p* < 0.05 was considered statistically significant, and the experimental data are presented as the means ± standard deviations.

## 3. Results

### 3.1. Frog Skin AMP 3-13 Attenuates AS in ApoE^-/-^ Mice by Regulating Cholesterol Metabolism

**Figure 1:**
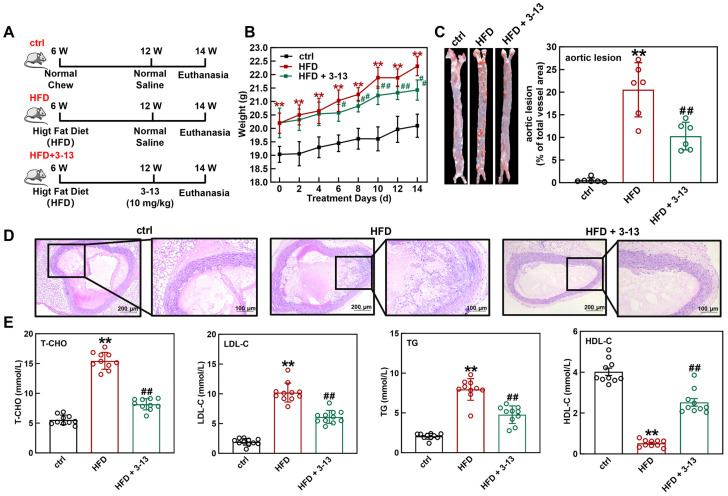
Frog skin AMP 3-13 alleviated AS in *ApoE^-/-^* mice by regulating cholesterol metabolism. (**A**) Experimental design flowchart during the experimental period. (**B**) Effects of the AMP 3-13 on body weight gain in *ApoE^-/-^* AS mice (*n* = 10). (**C**) ORO staining of the aortic vessels and quantification of the corrected plaque areas in *ApoE^-/-^* AS mice (*n* = 6). (**D**) H&E staining of the aortic root vessels in *ApoE^-/-^* AS mice. (**E**) Detection of T-CHO, LDL-C, TG and HDL-C levels in the plasma of *ApoE^-/-^* AS mice (*n* = 10). Statistical comparisons were carried out one-way ANOVA followed by Tukey post hoc. The data are presented as the means ± SDs. ** *p* < 0.01 vs. ctrl group, ^#^ *p* < 0.05, ^##^ *p* < 0.01 vs. HFD group.

To investigate the effect of AMP 3-13 on AS progression in vivo, a HFD-induced AS model in *ApoE^-/-^* mice was initially established. The genotype of *ApoE^-/-^* mice was detected via PCR and agarose gel electrophoresis. As shown in Figure S1, wild-type mice presented a DNA band at 155 bp, and *ApoE^-/-^* mice presented a band at 245 bp, verifying the successful generation of *ApoE^-/-^* mice. The experimental design flowchart was shown in Figure 1A. *ApoE^-/-^* mice were fed with HFD for 6 weeks and then treated with AMP 3-13 for 2 weeks. The body weight of the *ApoE^-/-^* mice in each group was recorded every day. As shown in Figure 1B, compared with the control group, the body weight of the *ApoE^-/-^* mice was increased by 1.16 ± 0.38 g after 6 weeks of HFD consumption. After 2 weeks of AMP 3-13 treatment, the body weight of the *ApoE^-/-^* mice in the control, HFD and AMP 3-13 groups was increased to 1.06 ± 0.43, 2.12 ± 0.35 and 1.22 ± 0.37 g, respectively, suggesting that AMP 3-13 treatment attenuated the weight gain induced by HFD.

Next, the effect of AMP 3-13 on atherosclerotic plaque formation in *ApoE^-/-^* AS mice was investigated. ORO staining of the aortic vessels was shown in Figure 1C. Compared with the control group, the aortic vessels in the HFD group presented larger areas of lipid-rich plaque. Compared with the HFD group, the plaque area in the aortic vessels was significantly reduced by 53.3% after 2 weeks of the treatment with AMP 3-13. Morphological changes in the aortic root vessels were also detected via H&E staining. As depicted in Figure 1D, the control group showed an intact lumen with neatly arranged and no plaque in the aortic root vessels. Conversely, the HFD group displayed markedly increased plaque deposition, whereas AMP 3-13 treatment significantly reduced atherosclerotic plaque formation in the aortic root, indicating its protective effect against HFD-induced AS in *ApoE^-/-^* mice.

The plasma T-CHO, LDL-C, TG and HDL-C contents are critical markers for evaluating the risk of AS. The contents of these lipids in mouse plasma were detected after the treatment with AMP 3-13. Compared with the control group, the plasma T-CHO, LDL-C and TG contents in the HFD group were increased by 64.1%, 81.1% and 74.0%, respectively, whereas the HDL-C content was decreased by 87.1% (Figure 1E). Moreover, AMP 3-13 decreased the T-CHO, LDL-C and TG contents by 47.1%, 40.2% and 40.0%, respectively, and increased the HDL-C content by 79.4% (Figure 1E). These results indicated that AMP 3-13 alleviated AS by regulating cholesterol metabolism in *ApoE^-/-^* mice.

### 3.2. Frog Skin AMP 3-13 Regulated the Gene Expression of Cholesterol Metabolism and PPAR Signaling Pathway in ApoE^-/-^ AS Mice

**Figure 2:**
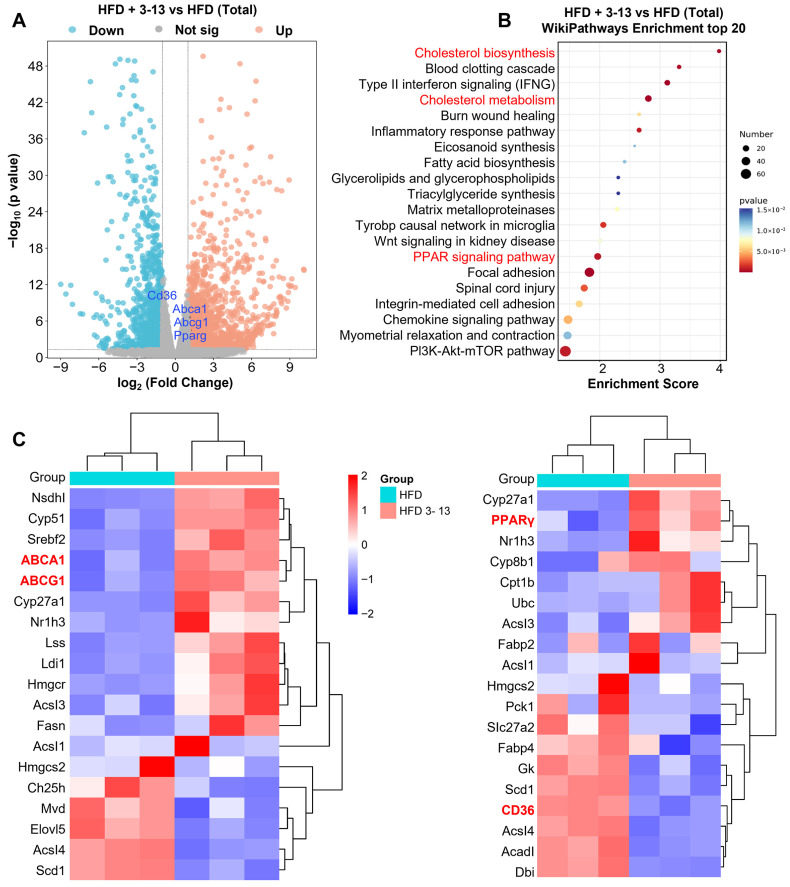
Transcriptome analysis of the effect of AMP 3-13 in the peritoneal macrophages of *ApoE^-/-^* AS mice. (**A**) Volcano plot of differentially expressed genes (DEGs) between *ApoE^-/-^* that were fed with a high-fat diet (HFD) with or without 3-13 treatment. Blue dots and red dots represented significantly downregulated and upregulated genes, and gray dots represented non-significant genes. (**B**) Wikipathways enrichment analysis of the DEGs between *ApoE^-/-^* AS mice with or without 3-13 treatment (top 20). (**C**) Heatmap of the DEGs in cholesterol metabolism and PPAR signaling pathway. Blue to red color scale indicates low to high expression levels. The red letters represent the significant DEGs and the Wikipathways Enrichment analysis.

To elucidate the anti-atherosclerotic mechanisms of AMP 3-13, transcriptome analysis was conducted to identify the differentially expressed genes (DEGs) of the peritoneal macrophages which were isolated from *ApoE^-/-^* mice with or without AMP 3-13 treatment. Transcriptomic profiling identified 1336 differentially expressed genes (DEGs) in the AMP 3-13-treated group compared to the HFD control group, using thresholds of |log2 fold change| > 1 and adjusted *p* < 0.05 (Benjamini–Hochberg method). Among these, 648 genes were upregulated and 688 were downregulated (Figure 2A). Functional enrichment analysis via WikiPathways revealed significant modulation of pathways associated with cholesterol synthesis, cholesterol metabolism, and PPAR signaling (Figure 2B). The heatmap of DEGs related with the key metabolic pathways was presented in Figure 2C. Notably, AMP 3-13 treatment led to a pronounced upregulation of key cholesterol efflux transporters, *ABCA1* (log_2_ FC = 1.21) and *ABCG1* (log_2_ FC = 1.16), within the cholesterol metabolism pathway. In the PPAR signaling pathway, *PPARγ* was upregulated (log_2_ FC = 1.17), while *CD36* was downregulated (log_2_ FC = −1.02), indicating a coordinated regulatory effect on lipid metabolism mediated by AMP 3-13.

**Figure 3:**
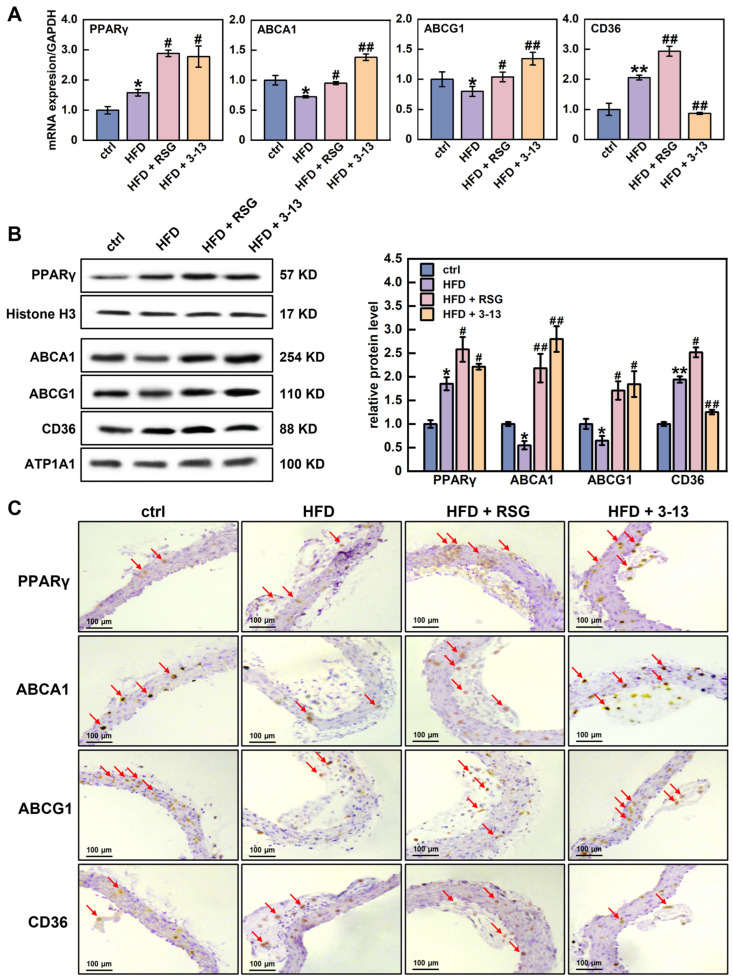
Effects of AMP 3-13 on the gene expression related with cholesterol metabolism and PPAR signaling pathway in *ApoE^-/-^* AS mice. (**A**) mRNA levels of *PPARγ*, *ABCA1*, *ABCG1* and *CD36* were detected by RT-qPCR (*n* = 6). (**B**,**C**) Protein levels of PPARγ, ABCA1, ABCG1 and CD36 were detected by Western blot (**B**) and immunohistochemistry (**C**, *n* = 6). Histone H3/ATP1A1 was used as an internal reference. Statistical comparisons were carried out one-way ANOVA followed by Tukey post hoc. The data are presented as the means ± SDs. * *p* < 0.05, ** *p* < 0.01 vs. ctrl group, ^#^ *p* < 0.05, ^##^ *p* < 0.01 vs. HFD group. Red arrow: representative positive result.

The expression of *PPARγ*, *ABCA1*, *ABCG1* and *CD36* genes in the aortic vessels was further detected by RT-qPCR and Western blot. RSG, a PPARγ agonist, was used as a positive control. RT-qPCR analysis showed that the mRNA levels of *PPARγ* and *CD36* were increased by 1.6- and 2.1-fold, respectively, and *ABCA1* and *ABCG1* were decreased by 27.7% and 20.2%, respectively, (Figure 3A). In addition, AMP 3-13 treatment further increased the mRNA levels of *PPARγ*, *ABCA1* and *ABCG1* by 2.8-, 1.4- and 1.3-fold, but decreased the mRNA level of *CD36* by 57.8% (Figure 3A).

Western blot results showed that PPARγ and CD36 protein levels were increased by 1.9-fold, whereas ABCA1 and ABCG1 protein levels were decreased by 45.3% and 35.1%, respectively, (Figure 3B). However, AMP 3-13 treatment increased PPARγ, ABCA1 and ABCG1 protein levels by 16.4%, 80.4% and 64.8%, respectively, but reduced CD36 protein level by 35.6% (Figure 3B). The Western blot results were also confirmed by immunohistochemistry. The results revealed that the protein expression of PPARγ and CD36 in the aortic root of the HFD group was increased, whereas the expression of ABCA1 and ABCG1 was decreased (Figure 3C). However, AMP 3-13 treatment reduced HFD-induced CD36 expression and increased PPARγ, ABCA1 and ABCG1 expression (Figure 3C). Several studies have reported PPARγ is a critical transcription factor involved in maintaining cellular cholesterol homeostasis. The activation of PPARγ upregulates *ABCA1*, *ABCG1* and *CD36* gene expression, thereby regulating cellular ox-LDL uptake and cholesterol efflux. The imbalance of ox-LDL uptake and cholesterol efflux is a crucial factor for foam cell formation. Therefore, we proposed that AMP 3-13 inhibited the formation of foam cells through the PPARγ-dependent cholesterol metabolism pathway, thereby exerting anti-atherosclerotic activity.

### 3.3. Frog Skin AMPs Inhibited Cholesterol Accumulation in THP-1-Derived Foam Cells

**Figure 4:**
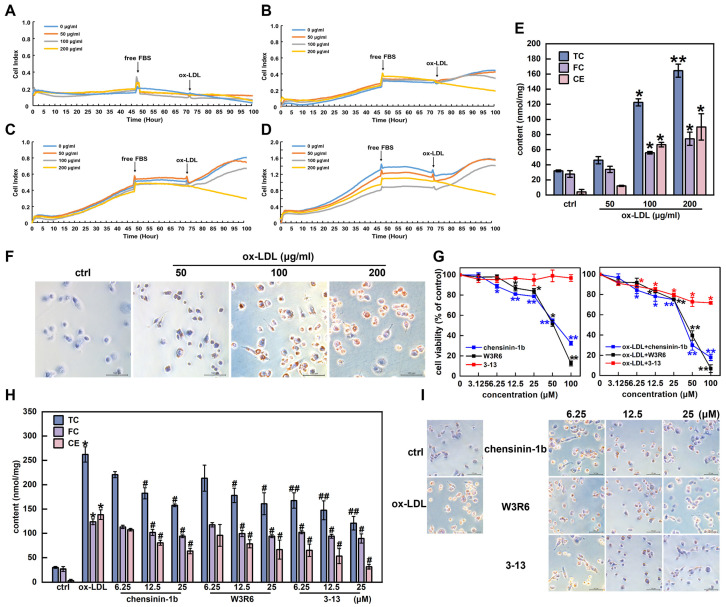
Frog skin AMPs inhibited cholesterol accumulation in THP-1-derived foam cells. (**A**–**D**) Effects of ox-LDL on the viability of THP-1 cells (**A**–**D**: 5 × 10^4^, 1 × 10^5^, 1.5 × 10^5^ and 2 × 10^5^ cells/mL). (**E**) Levels of total cholesterol (TC), free cholesterol (FC) and cholesterol ester (CE) in ox-LDL-induced foam cells. (**F**) Ox-LDL-induced changes in cell morphology and lipid droplet formation were analyzed via ORO staining (400×). (**G**) Effects of chensinin-1b, W3R6 and 3-13 on the viability of THP-1-derived macrophages and ox-LDL-induced foam cells. (**H**) Effects of AMPs on TC, FC and CE in ox-LDL-induced foam cells. (**I**) Analysis of the effects of AMPs on morphological changes and lipid droplet formation in ox-LDL-induced foam cells via ORO staining (400×). Statistical comparisons were carried out one-way ANOVA followed by Tukey post hoc. The data are presented as the means ± SDs. * *p* < 0.05, ** *p* < 0.01 vs. ctrl group, ^#^ *p* < 0.05, ^##^ *p* < 0.01 vs. ox-LDL group.

Chensinin-1b and W3R6 are analogs of 3-13, sharing similar structural characteristics [34]. The effects of the three AMPs on cholesterol metabolism in THP-1-derived foam cells were further explored. Initially, PMA-differentiated human THP-1 monocytes (M0 macrophages) were induced with ox-LDL to establish a foam cell model. To determine the proper cell density, the proliferation of THP-1 macrophages at different densities was monitored via RTCA. At a density of 5 × 10^4^ cells/mL, the proliferation rate of the THP-1 macrophages was relatively slow (Figure 4A). When the cell density was set to 1 × 10^5^ cells/mL, a notable increase in cell proliferation was observed (Figure 4B). At higher cell densities of 1.5 × 10^5^ and 2 × 10^5^ cells/mL, the cell proliferation began to decrease after 90 h (Figure 4C,D). Therefore, a THP-1 macrophage density of 1 × 10^5^ cells/mL was selected for subsequent experiments. The cytotoxicity of ox-LDL to THP-1 macrophages at concentrations of 50, 100 and 200 μg/mL was detected via RTCA. As shown in Figure 4A–D, ox-LDL treatment reduced the viability of THP-1 macrophages in a dose-dependent manner, with a rapid decrease in viability observed at 200 μg/mL. Therefore, 50 and 100 μg/mL were selected as the appropriate concentrations for inducing foam cell formation.

Foam cells are identified by abnormally abundant quantities of cholesterol and cholesteryl esters, with cholesterol esters accounting for more than 50% of the total cholesterol content [3]. As shown in Figure 4E, the TC and FC contents in THP-1 macrophages gradually increased with increasing ox-LDL concentrations. When the THP-1 macrophages were induced with ox-LDL at a concentration of 100 μg/mL, the levels of TC, FC, and CE increased by 73.9%, 50.3%, and 93.7%, respectively. When the ox-LDL concentration was increased to 200 μg/mL, the lipid content increased by 80.6%, 62.7%, and 95.4%, respectively. However, the lipid content was all less than 50%, with no significant difference in the 50 μg/mL ox-LDL group (*p* > 0.05). In addition, the CE/TC ratios of the 100 and 200 μg/mL ox-LDL groups reached 54.4% and 54.7%, respectively, meeting the criteria for inducing foam cell formation (CE/TC ratio above 50%). Furthermore, the ORO staining results revealed that the size and intracellular accumulation of lipid droplets significantly increased under ox-LDL treatment at concentrations greater than 50 μg/mL (Figure 4F). Considering the cytotoxicity of ox-LDL, a final concentration of 100 μg/mL was selected as the optimal concentration to induce foam cell formation.

The effects of the AMPs 3-13, W3R6 and chensinin-1b on the viability of THP-1 macrophages and ox-LDL-induced foam cells were subsequently detected via a CCK8 assay. As shown in Figure 4G, AMP 3-13 exhibited lower cytotoxicity to THP-1 macrophages and ox-LDL-induced foam cells than AMPs chensinin-1b and W3R6. When the concentrations of the three AMPs were below 25 μM, the AMPs did not display significant cytotoxicity to either of the cell types, with the cell viability remaining above 75%. Thus, 6.25, 12.5 and 25 μM were selected as the optimal concentrations of AMP 3-13 and its analogs for inhibiting ox-LDL-induced foam cell formation.

The effects of AMP 3-13 and its analogs on the cholesterol content in foam cells were detected. As shown in Figure 4H, compared with those in the control group, the TC, FC and CE contents in the ox-LDL group were increased by 88.6%, 78.4% and 97.7%, respectively, and the CE/TC ratio was 52.8%. However, compared with those in the ox-LDL group, the TC contents were decreased by 15.8–39.8%, 18.6–38.6% and 36.1–53.9% in the chensinin-1b, W3R6 and 3-13 AMP groups, respectively, the FC contents were decreased by 8.5–24.0%, 5.1–23.9% and 17.4–27.8%, respectively, and the CE contents were decreased by 22.3–54.0%, 30.7–51.8% and 52.9–77.3%, respectively. Notably, among the three AMPs, AMP 3-13 had the most significant inhibitory effect on the cholesterol content of foam cells. ORO staining revealed similar results. The number of intracellular lipid droplets in the ox-LDL group was significantly greater than that in the control group. Compared with the ox-LDL group, the three AMP groups exhibited a gradual reduction in the number of lipid droplets in foam cells (Figure 4I). These results indicated that AMP 3-13 and its analogs inhibited foam cell formation by reducing the accumulation of cholesterol and lipid droplets, with AMP 3-13 demonstrating the strongest inhibitory effect.

### 3.4. Frog Skin AMPs Enhance Cholesterol Efflux Through PPARγ-Dependent ABCA1/ABCG1 Activation

**Figure 5:**
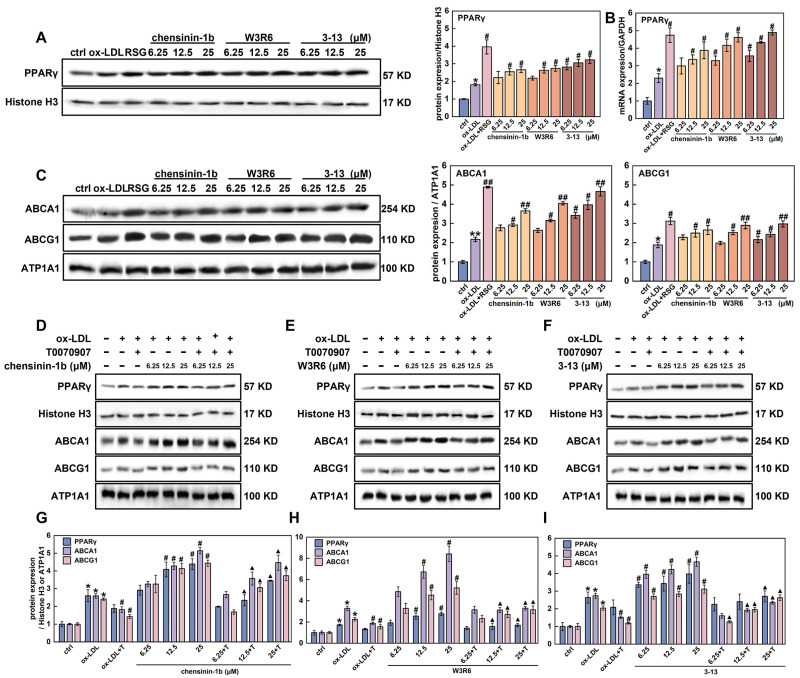
Frog skin AMPs upregulated the expression of PPARγ in foam cells. (**A**,**B**) Protein (**A**) and mRNA (**B**) expression of PPARγ in foam cells were detected by Western blot and RT-qPCR. (**C**) Protein expression of ABCA1 and ABCG1 in foam cells treated with frog skin AMPs was analyzed by Western blot. (**D**–**F**) Protein expression of PPARγ, ABCA1 and ABCG1 in foam cells that were treated with T0070907 in combination with chensinin-1b (**D**), W3R6 (**E**) or 3-13 (**F**) was analyzed by Western blot. (**G**–**I**) Quantitative statistical analysis of the protein expression levels of PPARγ, ABCA1 and ABCG1 in foam cells that were treated with T0070907 in combination with chensinin-1b (**G**), W3R6 (**H**) or 3-13 (**I**). Statistical comparisons were carried out one-way ANOVA followed by Tukey post hoc. The data are presented as the means ± SDs. * *p* < 0.05, ** *p* < 0.01 vs. ctrl group, ^#^ *p* < 0.05, ^##^ *p* < 0.01 vs. ox-LDL group. ^▲^ *p* < 0.05 vs. T0070907 (T) group.

PPARγ is a critical transcription factor involved in maintaining cellular cholesterol homeostasis and foam cell formation [40]. Therefore, we detected whether the three frog skin AMPs regulated PPARγ expression via Western blot and RT-qPCR in foam cells. As shown in Figure 5A, ox-LDL increased the PPARγ protein expression by 1.8-fold. Compared with the ox-LDL group, AMPs 3-13, W3R6 and chensinin-1b increased the protein expression by 18.1–31.8%, 17.0–33.7%, and 35.6–43.7%, respectively. In addition, ox-LDL increased *PPARγ* mRNA expression by 2.3-fold, and the three AMPs increased the mRNA expression by 72.0–79.6%, 69.6–78.3%, and 66.6–74.2%, respectively, (Figure 5B).

The activation of PPARγ upregulates ABCA1 and ABCG1 expression, thereby promoting cellular cholesterol efflux [41]. The effects of the three AMPs on the expression of ABCA1and ABCG1 were further detected via Western blot and RT-qPCR. As expected, ox-LDL increased ABCA1 and ABCG1 protein levels by 2.2-and 1.9-fold, respectively, (Figure 5C). Compared with the ox-LDL group, AMP 3-13, W3R6 and chensinin-1b upregulated ABCA1 protein levels by 36.7–53.5%, 17.9–46.4% and 21.8–40.7%, and ABCG1 protein levels by 12.9–36.9%, 5.1–35.1% and 17.4–29.2%, respectively. The trends in the mRNA expression levels of ABCA1 and ABCG1 were consistent with the protein expression levels, as both showed similar upregulation in response to AMP 3-13, W3R6, and chensinin-1b (Figure S2). The Western blot and RT-qPCR results indicated that AMP 3-13 and its analogs increased ABCA1 and ABCG1 expression via PPARγ, which had a similar effect as that of RSG (an agonist of PPARγ).

To further detect the role of PPARγ in the regulation of ABCA1 and ABCG1 expression in response to AMP 3-13 and its analogs, foam cells were treated with T0070907 (a specific PPARγ inhibitor) combined with one of the three AMPs, respectively. The expression of ABCA1 and ABCG1 proteins was detected via Western blot. As shown in Figure 5D–I, in the cotreatment group, T0070907 counteracted the ability of 3-13 to induce ABCA1 and ABCG1 expression, leading to lower protein levels of ABCA1 and ABCG1 than those in the 3-13 alone treatment group. Compared with ox-LDL, AMP 3-13 increased the expression of ABCA1 and ABCG1 by 30.7–41.2% and 24.3–34.5%, respectively, as the concentration increased from 6.25 to 25 μM. However, compared with 3-13 treatment alone at the corresponding concentrations, T0070907 decreased the expression of ABCA1 and ABCG1 by 49.6–59.5% and 15.7–53.2%, respectively, (Figure 5F,I). T0070907 had a similar inhibitory effect on ABCA1 and ABCG1 expression in the AMPs W3R6 and chensinin-1b groups as that was observed in the AMP 3-13 group (Figure 5D–I). The inactivation of PPARγ attenuated the upregulation of ABCA1 and ABCG1 induced by AMP 3-13 and its analogs, indicating that the AMPs regulated ABCA1 and ABCG1 expression via the PPARγ-mediated pathway.

ABCA1 and ABCG1 promote cellular cholesterol efflux and prevent excessive intracellular cholesterol aggregation. Therefore, the effects of the three AMPs on cholesterol efflux were detected by adding NBD-labeled cholesterol and simultaneously adding apoA-1 or HDL to mediate cholesterol efflux. As shown in Figure S3, in the absence of exogenous apoA-1 or HDL, the cholesterol efflux rate was approximately 17.5% in foam cells. After adding apoA-1 or HDL, the intracellular cholesterol efflux rate was increased to 21.8% or 40.5%, respectively. Compared with the apoA-1 or HDL group, AMP 3-13 further increased the cholesterol efflux rate by 28.9–35.2% or 8.7–18.4%, AMP W3R6 increased it by 7.3–25.2% or 16.0–28.7%, and AMP chensinin-1b increased it by 5.6–19.2% or 7.2–22.3%, respectively. These results indicated that AMP 3-13 and its analogs promoted cholesterol efflux by activating PPARγ-ABCA1/ABCG1 signaling pathway.

### 3.5. Frog Skin AMPs Inhibit ox-LDL Uptake by Competing with PPARγ to Bind with the CD36 Promoter

**Figure 6:**
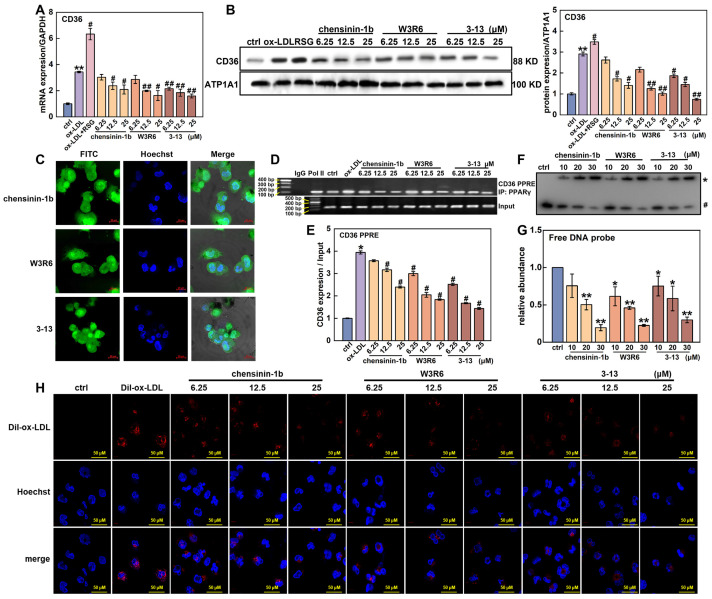
Frog skin AMPs downregulated CD36 expression by competing with PPARγ to bind with the *CD36* promoter. (**A**,**B**) mRNA and protein expression of CD36 in foam cells treated with frog skin AMPs was analyzed by RT-qPCR and Western blot. (**C**) The localization of AMP was analyzed by confocal laser scanning microscope. (**D**) Effects of AMPs on the binding ability between PPARγ and the *CD36* promoter were detected by ChIP assay. (**E**) Quantitative statistical analysis of the ChIP assay. Statistical comparisons were carried out one-way ANOVA followed by Tukey post hoc. The data are presented as the means ± SDs. * *p* < 0.05, ** *p* < 0.01 vs. ctrl group, ^#^ *p* < 0.05, ^##^ *p* < 0.01 vs. ox-LDL group. (**F**) The interaction between the AMPs and PPRE probes was analyzed by EMSA. * AMPs–DNA probe complex. # Free DNA probe. (**G**) Quantitative statistical analysis of the EMSA, * *p* < 0.05, ** *p* < 0.01 vs. ctrl group. (**H**) Impact of AMPs on Dil-ox-LDL uptake in foam cells was analyzed by fluorescence microscope.

PPARγ also plays a crucial role in the regulation of CD36 expression. It forms a heterodimer with RXRα, which binds to specific DNA sequences called PPRE, thereby promoting the transcription of CD36 [11,12,42]. The effects of the three AMPs on CD36 expression were detected via RT-qPCR and Western blot. As shown in Figure 6A, the mRNA level of *CD36* in the ox-LDL group was increased by 3.4-fold compared to the control group. However, when the concentration of AMP 3-13, W3R6, and chensinin-1b was increased from 6.25 to 25 μM, these AMPs effectively reduced *CD36* mRNA expression by 37.2–53.6%, 16.5–52.5%, and 11.7–39.0%, respectively. The Western blot data revealed a similar effect of these AMPs on the protein expression of CD36. As shown in Figure 6B, ox-LDL upregulated CD36 protein levels by 2.9-fold. However, AMPs 3-13, W3R6 and chensinin-1b downregulated CD36 protein levels by 35.9–74.5%, 25.7–65.2%, and 9.9–51.7%, respectively. Intriguingly, AMP 3-13 and its analogs upregulated the expression of PPARγ, although downregulated CD36 expression. In addition, compared with the three AMPs treatment alone at the corresponding concentrations, T0070907 (a PPARγ inhibitor) further decreased CD36 expression by 28.8–53.8%, 21.1–37.4% and 25.0–52.1%, respectively, (Figure S4). The inactivation of PPARγ further decreased the downregulation of CD36 that was induced by the three AMPs, demonstrating that the AMPs decreased CD36 expression through PPARγ.

To explain the paradox that AMP 3-13 and its analogs upregulated PPARγ expression but decreased the expression of CD36, the effects of the three AMPs on the binding ability between PPARγ and the *CD36* promoter were investigated. First, AMP 3-13, W3R6 and chensinin-1b were labeled with FITC, and their localization was detected via a confocal laser scanning microscope. As shown in Figure 6C, AMP 3-13 and its analogs were distributed not only in the cytoplasm but also in the nucleus, particularly in the nucleolus. Among these three AMPs, AMP 3-13 exhibited the highest distribution in the nucleus. The nuclear location of these AMPs indicated their potential to disrupt the interaction between PPARγ and the *CD36* promoter. Next, the effects of the AMPs on *CD36* promoter activity were detected via a dual-luciferase reporter assay. As shown in Figure S5, compared with the control group, the overexpression of PPARγ increased the activity of the *CD36* promoter (including a PPRE motif) by 4.8-fold, whereas AMPs 3-13, W3R6 and chensinin-1b reduced the activity of the *CD36* promoter by 22.9–57.2%, 24.0–57.8% and 25.3–56.2%, respectively, as the concentration increased from 6.25 to 25 μM. In addition, the effects of the three AMPs at 6.25 μM did not significantly differ from those of the control group (*p* > 0.05); however, significant differences were observed at higher concentrations. These results demonstrated that AMP 3-13 and its analogs reduced the activity of the *CD36* promoter. Furthermore, the effects of these AMPs on the binding ability of PPARγ to the *CD36* promoter were detected via a ChIP assay. As shown in Figure 6D,E, compared with that in the IgG-ChIP-negative group, the DNA fragment containing the PPRE motif in the *CD36* promoter was significantly enriched in the PPARγ-ChIP group, confirming that PPARγ targeted to the *CD36* promoter. In addition, ox-LDL treatment increased the enrichment of the DNA fragment containing the PPRE motif by 3.9-fold. However, compared with the ox-LDL group, AMP 3-13 and its analogs decreased the enriched DNA levels by 36.1–63.6%, 24.0–53.4% and 9.4–39.2%, respectively, indicating that the AMPs decreased the binding ability between PPARγ and the *CD36* promoter. The interaction between the three AMPs and PPRE probes was examined via EMSA. As the concentration of AMP 3-13 and its analogs increased, the amount of free PPRE probes was decreased (Figure 6F,G). The EMSA data further confirmed that AMP 3-13 and its analogs directly bound to the PPRE motif.

CD36 acts as the major scavenger receptor to ox-LDL uptake in macrophages. Therefore, the effects of the frog skin AMPs on Dil-ox-LDL uptake were visualized via fluorescence microscopy. Compared with the control group, the intracellular fluorescence signal notably increased in the Dil-ox-LDL group (Figure 6H), indicating that a substantial amount of Dil-ox-LDL was taken up by foam cells. However, the three AMPs rapidly reduced the fluorescence signal of Dil-ox-LDL, and the inhibitory effect became stronger as the AMP concentration increased. These results demonstrated that 3-13 and its analogs downregulated CD36 expression by competing with PPARγ to bind with the CD36 promoter, thereby inhibiting ox-LDL uptake in foam cells.

## 4. Discussion

Atherosclerosis is the primary risk factor for cardiovascular disease, which is the leading cause of morbidity and mortality in developed countries, and is becoming increasingly widespread in developing countries [1,43,44]. Foam cell formation is a critical event in the early stages of AS, which are characterized by abnormally high levels of cholesterol and cholesteryl esters [2,3,45]. Inhibiting foam cell formation is crucial for developing therapeutic strategies to prevent and treat AS [13]. For instance, LL-37 has been shown to mitigate cholesterol accumulation by downregulating CD36 expression through the ERK signaling pathway [29]. Additionally, PR-39 exerts cardioprotective effects by inhibiting proteasome-mediated degradation of IκBα, thereby suppressing inflammatory responses [31]. Our study previously demonstrated that the frog skin peptide temporin-1CEa and its analogs inhibited ox-LDL uptake in foam cells by downregulating CD36 expression, consequently ameliorating the accumulation of cholesterol within cells [32]. In this study, three frog skin AMPs 3-13, W3R6 and chensinin-1b, were designed and synthesized on the basis of the parent peptide chensinin-1, a natural peptide that was isolated from *Rana chensinensis*. To investigate the effects of these AMPs on AS, we utilized *ApoE^-/-^* mice fed a high-fat diet (HFD) to establish an AS mouse model and induced THP-1 cells with ox-LDL to create a foam cell model. Here, we revealed that AMP 3-13 and its analogs alleviate AS through a dual mechanism: activating the PPARγ-ABCA1/ABCG1 signaling pathway to promote cholesterol efflux, and inhibiting CD36-dependent ox-LDL uptake by competitively binding to the *CD36* promoter, thereby disrupting PPARγ-mediated transcriptional activation.

Atherosclerotic plaque formation is primarily driven by dysregulated cholesterol metabolism. To evaluate the therapeutic potential of AMP 3-13, we first examined its effects in *ApoE^-/-^* AS mice. The results demonstrated that AMP 3-13 significantly decreased weight gain, aortic root plaque formation, and the plasma cholesterol levels in *ApoE^-/-^* AS mice, suggesting that the AMP might alleviate AS by regulating cholesterol metabolism in *ApoE^-/-^* mice. To investigate the effect of AMP 3-13 on the regulation of AS, transcriptome was performed to analyze the differentially expressed genes in the peritoneal macrophages, which were isolated from *ApoE^-/-^* AS mice following treatment with or without AMP 3-13. Wikipathways enrichment analysis revealed that AMP 3-13 treatment regulated the gene expression related with cholesterol metabolism and the PPAR signaling pathway, with notable changes in the expression of PPARγ, ABCA1, ABCG1 and CD36. Notably, PPARγ is a critical transcription factor involved in maintaining cellular cholesterol homeostasis and foam cell formation. Previous studies have suggested an important role of the PPARγ-LXRα-ABCA1/ABCG1 pathway in regulating cholesterol efflux, which could be a promising target for treating AS [17,46]. For example, mangiferin, a xanthonoid from *Salacia oblonga*, promotes macrophage cholesterol efflux and protects against AS by upregulating ABCA1 and ABCG1 expression [17]. Additionally, Bu1 and Bu2 peptides inhibit foam cell formation by activating the PPARγ/LXRα signaling pathway and promoting cholesterol efflux [46]. In this study, AMP 3-13 and its analogs increased the expression of PPARγ, ABCA1 and ABCG1 in foam cells. In addition, a PPARγ inhibitor (T0070907) attenuated the increased expression of ABCA1 and ABCG1 induced by AMP 3-13 and its analogs, indicating that these AMPs increased the expression of ABCA1 and ABCG1 via the PPARγ-mediated pathway to promote cholesterol efflux.

It has been reported that PPARγ plays a crucial role in regulating ox-LDL uptake via CD36 [42]. Mechanically, PPARγ binds with RXRα to form heterodimers, which interact with the PPRE motif in the *CD36* promoter [11,12]. Inactivation of the PPARγ/CD36 signaling pathway is a potential strategy for treating AS [47,48,49]. Tamoxifen, a selective estrogen receptor modulator, inhibits CD36 expression and cellular ox-LDL accumulation by downregulating PPARγ and disrupting the interaction between PPARγ and PPRE in the *CD36* promoter [47]. In addition, numerous natural products, such as black mulberry ethanol extract and spiromastixones, exert anti-AS effects by inhibiting PPARγ and CD36 expression [48,49]. Our results revealed that AMP 3-13 and its analogs upregulated PPARγ expression but downregulated CD36 expression. We proposed that AMPs might disrupt the binding ability of PPARγ to the *CD36* promoter. This hypothesis was verified via dual-luciferase reporter gene and ChIP assays. The results demonstrated that AMP 3-13 and its analogs were localized in the nucleus, particularly in the nucleolus, suggesting their potential to interfere with the binding of PPARγ to the *CD36* promoter. The dual-luciferase reporter gene results revealed that AMP 3-13 and its analogs reduced the activity of the *CD36* promoter. The ChIP results indicated that the AMPs decreased the binding ability of PPARγ to the *CD36* promoter. The EMSA results further demonstrated that the AMPs bound directly to the PPRE motif.

The mechanisms of canonical AMPs primarily involve membrane disruption or intracellular targeting to disrupt protein–protein interactions. Direct nuclear localization and competitive interference with specific transcription factor–DNA interactions represent a less conventional mechanism for AMPs. However, there are some examples of stapled peptides that disrupt protein–DNA interactions. For instance, hydrocarbon-stapled peptides competitively bind to Nrf2 response elements, thereby inhibiting Nrf2–DNA interactions and suppressing Nrf2 transcriptional activation in cancer cells [50,51]. Similarly, a stapled peptide based on GCN4 retains DNA-binding ability and enhancing cellular uptake [52,53]. Although AMP 3-13 and stapled peptides demonstrate structural differences, they share common physicochemical properties including a high density of cationic amino acids and a strong propensity for α-helix formation. These conserved features likely contribute to competitively inhibit DNA binding to transcription factor domains. This suggests a novel peptide mechanism involving direct modulation of transcriptional complexes at genomic regulatory elements.

However, several important limitations must be acknowledged. Firstly, despite our results demonstrated that AMP 3-13 and its analogs bound directly to the PPRE motif and decreased the binding ability of PPARγ to the *CD36* promoter, we could not definitively conclude whether the observed AMP-PPRE interaction represents a sequence-specific recognition event or stems from non-specific electrostatic interactions. Electrostatic interactions between the negatively charged DNA and positively charged regions of the protein play a role in these non-specific DNA–protein interactions [54]. Due to its charge properties, the binding of AMPs to DNA probably represents non-specific DNA–protein interactions. However, it cannot totally explain for the results in our study for the following reasons: (a) AMP 3-13 and its analogs treatment led to a significant upregulation of PPARγ expression while significantly downregulation of CD36 expression. If AMPs bound with DNA through non-specific manner, a broader suppression of gene expression would be observed. Notably, we found that the specific suppression of CD36, while the expression levels of ABCA1 and ABCG1, which are also downstream targets of PPARγ, are upregulated. These results suggested a mechanism involving intervention at specific promoter responsive element rather than a global, non-specific block. (b) AMP 3-13 and its analogs demonstrated strictly concentration-dependent effects on PPRE-binding affinity (EMSA, Figure 6F), and the interaction between PPARγ and the *CD36* promoter (ChIP, Figure 6D,E). Although non-specific binding interactions may exhibit limited concentration dependence, they generally fail to produce the precisely calibrated, target-specific dose–response relationships observed in our study. (c) Furthermore, EMSA results (Figure 6F) showed that AMP 3-13 and its analogs binding to the target promoter probe formed discrete bands with specific mobility shifts, rather than diffuse smearing or precipitation. Although this did not entirely rule out non-specificity, the presence of discrete bands was generally more indicative of ordered binding or complex formation, rather than purely random, non-specific binding. Collectively, while non-specific DNA binding may contribute to initial AMPs interactions, the ChIP and EMSA results indicated a model of specific competitive interference at PPARγ/CD36 promoter regions. The definitive proof of sequence-specific recognition between AMPs and PPRE would require structural studies (e.g., crystallography) or binding affinity assays comparing mutated PPRE sequences. Elucidating the precise mode of action will be a key focus of our future research. Secondly, the therapeutic potential of AMPs is currently constrained by limited safety assessment. Preliminary hepatic histology showed preserved liver architecture without necrosis or inflammation at the tested dose (10 mg/kg, 2 weeks; Figure S6). However, comprehensive safety assessment including renal histopathology and serum biochemistry was not performed; the complete toxicity profile is to be established in the future.

## 5. Conclusions

**Figure 7:**
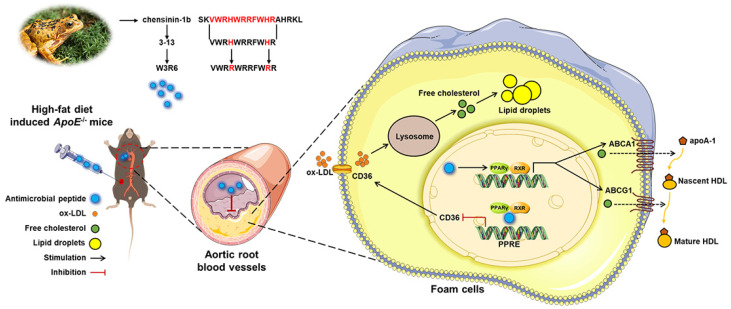
Schematic representation of the mechanism by which AMP 3-13 and its analogs alleviate atherosclerosis. AMP 3-13 significantly alleviated aortic root plaque formation in *ApoE^-/-^* AS mice and reduced cholesterol accumulation in THP-1-derived foam cells. AMP 3-13 and its analogs activated PPARγ-ABCA1/ABCG1 to promote cholesterol efflux, whereas the AMPs competed with PPARγ to bind with the *CD36* promoter to inhibit ox-LDL uptake.

In conclusion, this study demonstrated that AMP 3-13 and its analogs attenuated AS both in vitro and in vivo. In *ApoE^-/-^* AS mice, the AMP 3-13 significantly decreased weight gain, aortic root plaque formation, and plasma cholesterol levels to ameliorate AS. Moreover, AMP 3-13 significantly increased the expression of PPARγ, ABCA1 and ABCG1 but decreased the expression of CD36 in aortic root vessels, thereby regulating cholesterol metabolism. In addition, the three AMPs suppressed foam cell formation through dual mechanisms: promoting cholesterol efflux by activating the PPARγ-ABCA1/ABCG1 signaling pathway and preventing ox-LDL uptake by disrupting the interaction of PPARγ and the *CD36* promoter (Figure 7). Among AMP 3-13 and its analogs, AMP 3-13 had the most significant inhibitory effect on the cholesterol content in foam cells. This study demonstrated promising prospects for AMP 3-13 and its analogs as therapeutic agents for the prevention and treatment of AS.

## Data Availability

Data are available upon request to the corresponding author.

## Supplementary Materials

The following supporting information can be downloaded at https://www.mdpi.com/article/10.3390/cells14181470/s1, Figure S1: Genetic identification of *ApoE^-/-^* mice via agarose gel electrophoresis; Figure S2: mRNA expression of *ABCA1* and *ABCG1* was detected by RT-qPCR; Figure S3: Impact of AMPs on NBD-labeled cholesterol efflux rates in the presence of exogenous apoA-1 or HDL in foam cells; Figure S4: Protein expression of CD36 in foam cells that were treated with T0070907 in combination with AMPs was analyzed by Western blot; Figure S5: The binding ability between PPARγ and the CD36 promoter was analyzed by dual-luciferase reporter gene assay; Figure S6: Toxicity of AMP 3-13 in *ApoE^-/-^* AS mice; Table S1: Physicochemical information of the frog skin AMPs; Table S2: Primers for RT-qPCR; Table S3: Antibody for Western Blot and Immunohistochemistry.

## Abbreviations

The following abbreviations are used in this manuscript:

ABCA1/G1	ATP binding box transporter A1/G1
AMPs	Antimicrobial peptides
apoA-1	Apolipoprotein A-1
ApoE^-/-^	Apolipoprotein E gene knockout
AS	Atherosclerosis
CD36	Cluster of differentiation 36
CE	Cholesteryl ester
ChIP	Chromatin immunoprecipitation
FC	Free cholesterol
HDL	High-density lipoprotein
HFD	High-fat diet
LDL-C	Low-density lipoprotein cholesterol
NBD	Nitrobenzoxadiazole
ORO	Oil red oxygen
ox-LDL	Oxidized low-density lipoprotein
PPARγ	Peroxisome proliferator-activated receptor γ
PPRE	Peroxisome proliferator-responsive element
RSG	Rosiglitazone
TC	Total cholesterol
TG	Triglyceride
