# Supplementary material for: Frog Skin Antimicrobial Peptide 3-13 and Its Analogs Alleviate Atherosclerosis Cholesterol Accumulation in Foam Cells via PPARγ Signaling Pathway
Source: Cells. 2025 Sep 19;14(18):1470. doi: 10.3390/cells14181470 (PMC12468074; doi:10.3390/cells14181470)
Supplement: Supplementary file 1 [file cells-14-01470-s001.zip › cells-3753893-supplementary.pdf]

Table S1 Physicochemical information of the frog skin antimicrobial peptides

Antimicrobial Peptides	Sequence	Molecular Weight	Hydrophobic Value	Charge Number
chensinin-1b	SKVWRHWRRFWHRAHRKL	2542.9	9.69	+7
W3R6	VWRRWRRFWRR	1760.1	13.03	+6
3-13	VWRHWRRFWHR	1722.1	12.92	+4

Table S2 Primers for RT-qPCR

Primers	Sequence	Gene Serial Number
PPARγ	5'-ACCAAAGTGCAATCAAAGTGGA-3'<br>5'-ATGAGGGAGTTGGAAGGCTCT -3'	NM_001330615.4
ABCA1	5'-CCTGAAGCCAATCCTGAGAACAC-3'<br>5'-ACCTCCTGTCGCATGTCACTC-3'	NM_005502.4
ABCG1	5'-GAACTGCCCAACCTACCACAACC-3'<br>5'-GCCCTCCCGAACCGCTCTC-3'	NM_016818.3
CD36	5'-AACAGCAGCAACATTCAAGTTAAGC-3'<br>5'-GTCCTCAGCGTCCTGGGTTAC-3'	NM_000072.3
GAPDH	5'-GTGGACCTGACCTGCCGTCTAG-3'<br>5'-GAGTGGGTGTCGCTGTTGAAGTC-3'	NM_001289745.3

Table S3 Antibody for Western Blot and Immunohistochemistry

Antibody	Manufacturer	Cat No.	Assay
PPARγ	Abclonal	A0270	WB & IHC
Histone H3	Abclonal	A2348	WB
ABCA1	Abclonal	A22125	WB
ABCG1	Abclonal	A17907	WB
CD36	Abclonal	A5792	WB & IHC
ATP1A1	Proteintech	14418-1-AP	WB
ABCA1	Abclonal	A25231	IHC
ABCG1	Abclonal	A4328	IHC

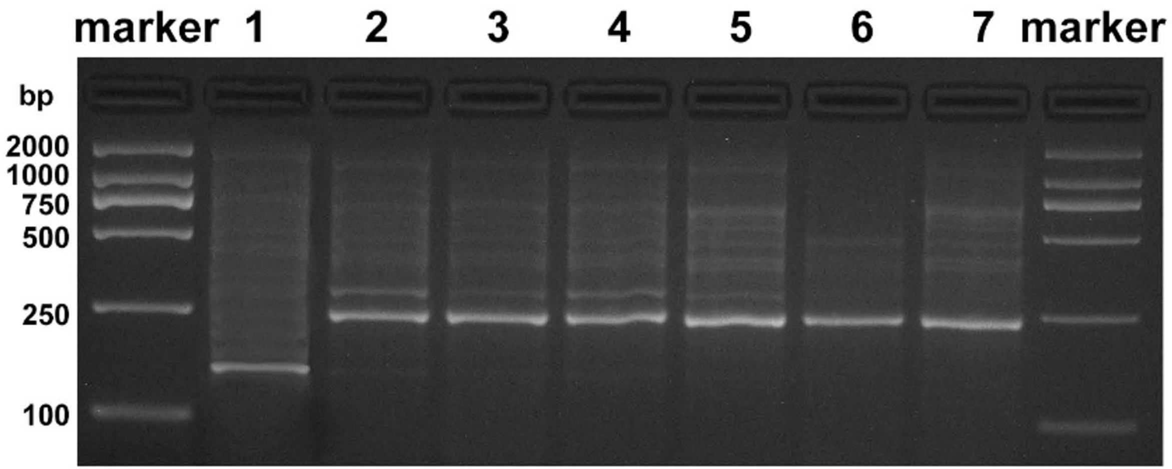

Figure S1 Genetic identification of *ApoE*$^{-/-}$ mice via agarose gel electrophoresis (Lane 1: wild-type mice; Lanes 2-7: *ApoE*$^{-/-}$ mice).

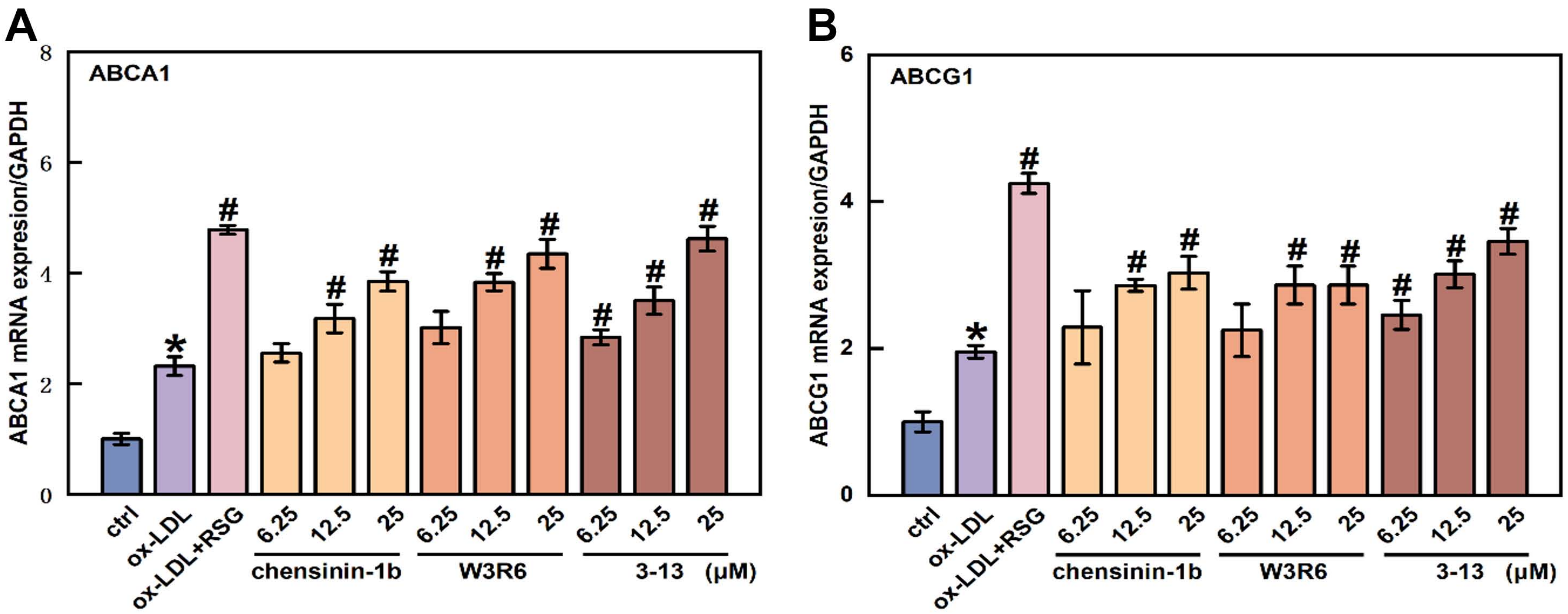

Figure S2 mRNA expression of ABCA1 **(A)** and ABCG1 **(B)** was detected by RT-qPCR (n=6). *p<0.05 ox-LDL group vs. control (ctrl) group, #p<0.05 ox-LDL+AMP group vs. ox-LDL group,

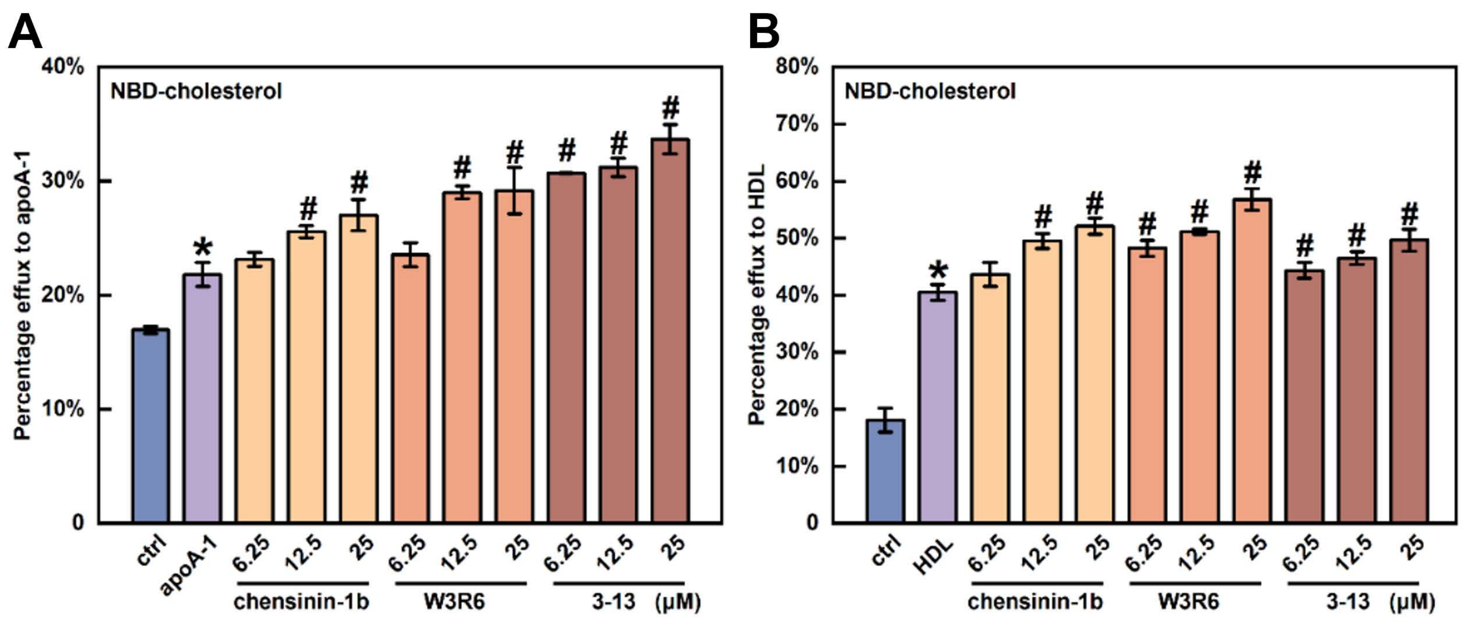

Figure S3 Impact of AMPs on NBD-labeled cholesterol efflux rates in the presence of exogenous apoA-1 **(A)** or HDL **(B)** in foam cells. *$p<0.05$ vs. the control (ctrl) group, #$p<0.05$ vs. the apoA-1 or HDL group.

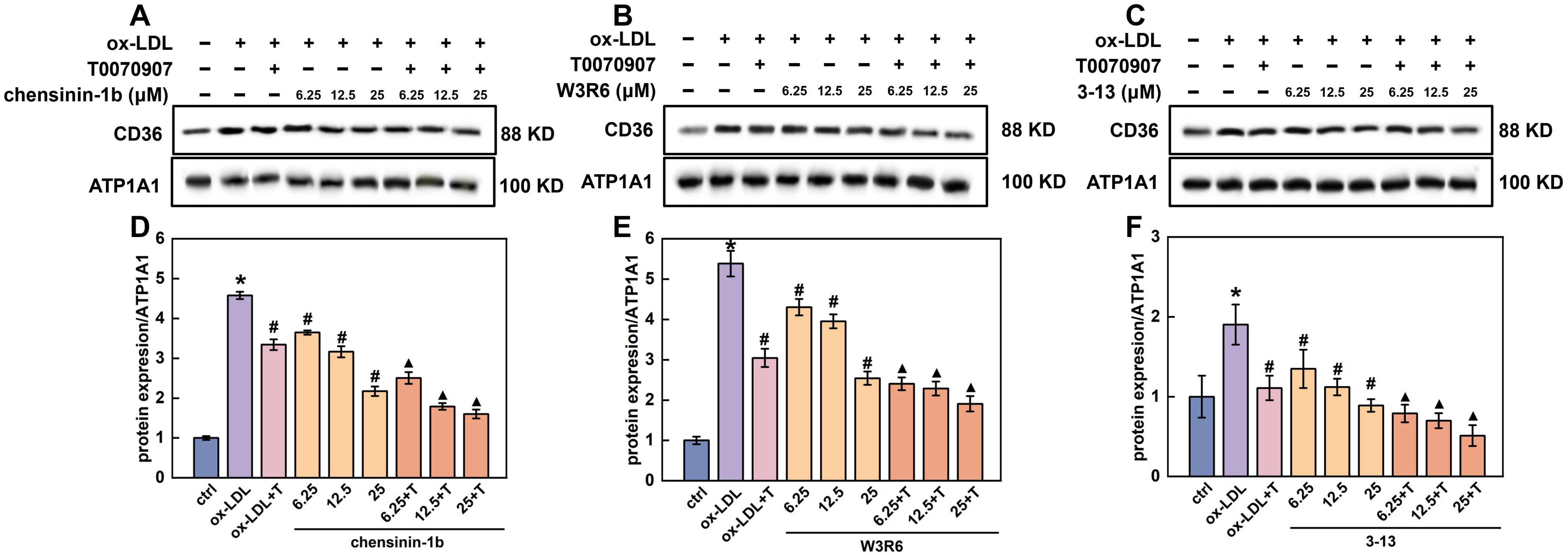

Figure S4 A-C Protein expression of CD36 in foam cells that were treated with T0070907 in combination with chensinin-1b **(A)**, W3R6 **(B)** or 3-13 **(C)** was analyzed by Western blot. **D-F** Quantitative statistical analysis of the protein expression levels of CD36 in foam cells that were treated with T0070907 in combination with chensinin-1b **(D)**, W3R6 **(E)** or 3-13 **(F)**.*p<0.05 ox-LDL group vs. control (ctrl) group, #p<0.05 ox-LDL+AMP group vs. ox-LDL group, ▲p<0.05 ox-LDL+AMP group vs. T0070907 (T) group.

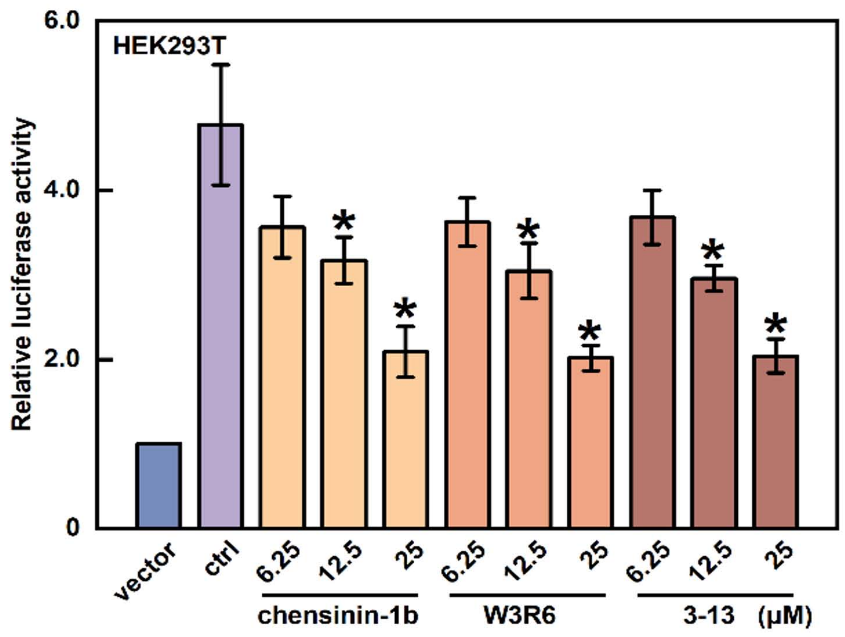

Figure S5 The binding ability between PPARγ and the CD36 promoter was analyzed by dual-luciferase reporter gene assay.*p<0.05 AMP group vs. ctrl.

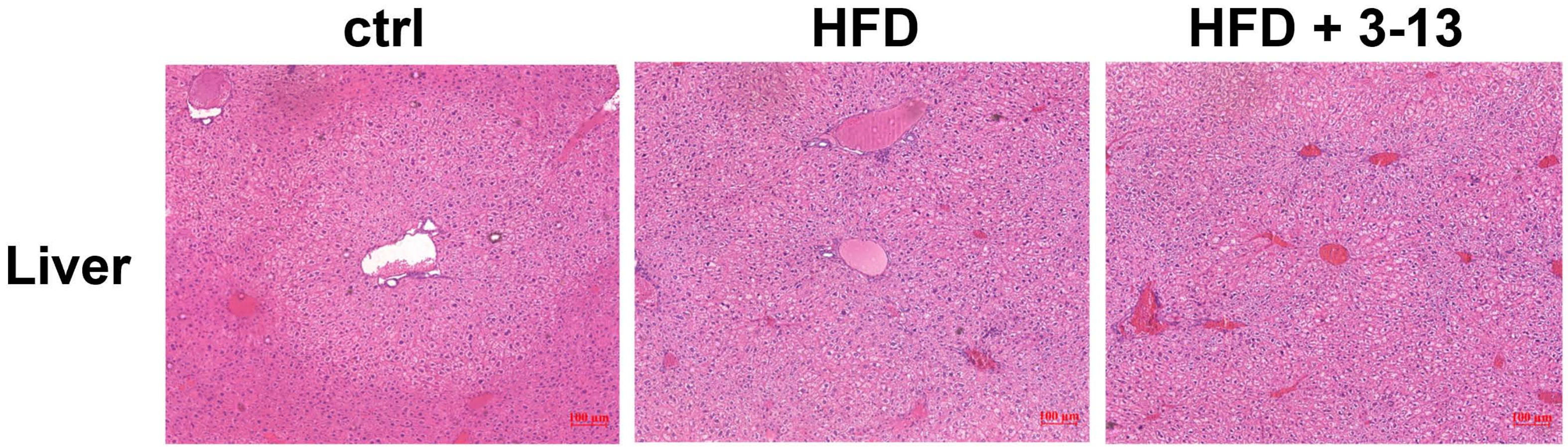

Figure S6 Toxicity of AMP 3-13 in *ApoE*$^{-/-}$ AS mice. H&E analysis of liver of *ApoE*$^{-/-}$ AS mice after the treatment with AMP 3-13 at the concentration of 10 mg/kg for 2 weeks (n=6).
